# Resource Sharing between the Invasive *Sirex noctilio* and Native Woodborers and Beetles in *Pinus* Plantations

**DOI:** 10.3390/insects15070478

**Published:** 2024-06-27

**Authors:** Ming Wang, Chenglong Gao, Ningning Fu, Lili Ren, Youqing Luo

**Affiliations:** 1 Beijing Key Laboratory for Forest Pest Control, Beijing Forestry University, Beijing 100083, China; 13020028768@163.com (M.W.); gaocl890907@163.com (C.G.); funingning2012@sina.com (N.F.); 2 Guangdong Key Laboratory of Animal Conservation and Resource Utilization, Guangdong Public Laboratory of Wild Animal Conservation and Utilization, Institute of Zoology, Guangdong Academy of Sciences, Guangzhou 510260, China; 3 Sino-French Joint Laboratory for Invasive Forest Pests in Eurasia, INRAE-Beijing Forestry University, Beijing 100083, China; 4 Guangdong Provincial Key Laboratory of Silviculture, Protection and Utilization, Guangdong Academy of Forestry, Guangzhou 510520, China; 5 Department of Forest Protection, College of Forestry, Hebei Agricultural University, Baoding 071033, China

**Keywords:** bark beetles, density, body size, resource utilization, *Sirex* woodwasp, woodborers

## Abstract

**Simple Summary:**

The invasive woodwasp *Sirex noctilio* occasionally shares hosts with the native *S. nitobei* and other colonizers. The coexistence of these species may significantly impact individual species. We compared coexistence patterns among several colonizers at both stand and tree scales. Spatial scales revealed negative associations (e.g., *Asemum striatum*, *Phaenops* sp.) and neutral ones (*Ips acuminatus*) between woodwasps and other co-colonizing insects. A positive correlation was found between the frequency of woodwasp attacks and the density of trees damaged by *Sirex* in the current and previous years. At the tree scale, *S. noctilio* is more abundant in sections where it occurs alone than in sections where it co-occurs with *S. nitobei*. The distribution and body size of *S. noctilio* within the tree are unaffected by the presence of *S. nitobei*. However, *S. nitobei* was more prevalent in bottom sections when *S. noctilio* was present and larger in bottom sections where it occurred alone. This result can be attributed to early *S. noctilio* attacks enhancing the micro-environment of *S. nitobei*, which provides nutrients for larval development.

**Abstract:**

*Sirex noctilio*, a European woodwasp, occasionally shares resources with the native *S. nitobei* and other colonizers in northeast China. The impact of its coexistence on individual species remains unclear. Random sampling was conducted to assess the patterns and extent of insect co-colonization across various spatial scales. Additionally, we analyzed wood sections to determine the density, adult size, and distribution of the two *Sirex* species. Spatial scales revealed negative associations (*Asemum striatum* and *Phaenops* sp.) and neutral ones (*Ips acuminatus*) between woodwasps and other co-colonizers. Clustering of woodwasps and *Phaenops* sp. occurred at a small scale (0–7.3 m). Regression analysis showed a positive correlation between the chance of woodwasp attacks and past attacks on the same host, with little impact from other colonization factors. The distribution and body size of *S. noctilio* within the tree appeared unaffected by *S. nitobei*’s presence. In the presence of *S. noctilio*, *S. nitobei* tended to lay eggs in damaged sections. At the stand level, the overall impact of *S. noctilio* on *S. nitobei* population density is likely positive because *S. nitobei* prefer weaker trees, a preference potentially influenced by initial attacks from *S. noctilio* on healthier hosts.

## 1. Introduction

Biological invasions are occurring at an unprecedented rate due to increased global trade and transportation, posing a threat to indigenous communities in invaded areas [1,2]. Species diversity amplifies the likelihood of interaction between sympatric invasive and native species, potentially influencing their ecology and evolution [3]. Within forest ecosystems, interactions (facilitative or antagonistic) among insects, whether direct or indirect, are common [4,5,6]. Primary bark beetles consume resin through collective attacks, which makes the tree more accessible to secondary pests and bark beetles [7]. For instance, pioneer species *Dendroctonus frontalis* Zimmermann renders hosts vulnerable, thus facilitating colonization by numerous secondary species [8]. The damage caused by *Agrilus planipennis* Fairmaire can temporarily provide a resource supply for local saproxylic insects and predators such as woodpeckers [9]. Furthermore, it can facilitate the expansion of the range of local buprestid beetle populations in Europe [10].

*Sirex noctilio* Fabricius, a woodwasp native to Europe, was first investigated in northeast China in 2013 [11]. Female *S. noctilio* drill eggs, symbiotic fungi, and toxins into the host tree [11,12,13]. The synergistic action of the fungus *Amylostereum areolatum* (Fr.) Boiden and phytotoxic mucus overwhelms the tree’s defense mechanisms and provides a suitable environment for larvae [14,15]. *Sirex noctilio* completes its generation development in 1–2 years, with most completing development within a year in China [16]. A 2015 field survey found sympatric distribution of *S. noctilio* and *S. nitobei* in northeast China [17,18]. Research shows that the Asian native *S. nitobei* (found in China, Japan, and Korea) has one generation per year and is associated with *A. areolatum* or *A. chailletii* (Pers.: Fr.) Boidin [12,19,20,21]. Direct competition is expected between two woodwasps as they develop in the sapwood, phloem, and xylem. These species may interact indirectly by inducing tree defenses, altering tree physiological state, or affecting tree decline, thus influencing nutrition or other resources. Based on the literature, *S. noctilio* prefers stressed trees, and it may even infest healthy trees [22,23]. In contrast, *S. nitobei* primarily prefers severely weakened or recently dead trees [24]. It can be concluded that the host niche width of *S. noctilio* is larger than that of *S. nitobei*. Considering that *S. noctilio* attacks healthy trees, which are later co-colonized by *S. nitobei* as the trees decline, *S. noctilio* likely contributes to *S. nitobei* at this level [24]. The extent to which the damage caused by *S. noctilio* may provide a habitat for *S. nitobei* is unclear. The sympatric distribution of two woodwasps in Northeast China provides sample sites to study their coexistence extent and impacts of their interaction.

Similar to the damage observed in North America [25,26], *S. noctilio* has encountered some native co-colonizers in Northeast China. *Ips acuminatus* (Gyllenhal, 1827) (Coleoptera: Curculionidae) is prominent among the diversity of scolytines that infest the interface between bark and xylem, while various woodborers (Cerambycidae and Buprestidae) burrow deep into the xylem tissue. Interactions among *Sirex* woodwasps, bark beetles, and woodborers are probably indirect [27,28,29], which can influence their distribution and abundance [30,31,32]. In North America, competition between native colonizers and *S. noctilio* might have restrained significant outbreaks of this invader [33]. Therefore, primary tree-attacking insects may experience negative effects from co-colonization with heterospecifics due to resource competition or intraguild predation [26,31,34,35,36]. Interactions between *S. noctilio* and other insects remain unexplored in its native European range due to its limited economic significance. Consequently, our understanding of these complex species interactions remains incomplete.

The temporal and spatial niches of *S. noctilio* and/or *S. nitobei* have been documented in certain regions (e.g., Dumeng (DM), Hegang (HG)) [17,37,38], but analyses of dispersal, colonization, as well as the abundance and quality of host materials are lacking for populations in Northeast China. Additionally, woodwasps are influenced not only by current-year colonizers but also by those from previous years. Here, we have specifically investigated the patterns of native *S. nitobei* and invasive *S. noctilio*, in mixed coniferous forests in Jinbaotun (JBT) and Yushu (YS). These forests represent the primary habitat for *S. nitobei* and *S. noctilio* since their introduction [12,17]. We examined colonizers contributing to pine mortality: *Sirex* species, bark beetles, and woodborers. Specifically, we aimed to determine the extent of co-occurrence of *Sirex* spp. and other competitors at the stand scale; evaluate the impacts of co-occurrence on body size and density at the scale of individual trees; and investigate resource partitioning in co-colonized trees through differential density based on trunk height.

## 2. Materials and Methods

### 2.1. Study Sites

The study was conducted in JBT and YS, which primarily consisted of 30-year-old plantations of *Pinus. sylvestris* var. *mongolica* Litv. and *P. tabuliformis* Carrière. Four locations were chosen for this study due to their documented history of significant infestations by *S. noctilio* and *S. nitobei* between the years 2017 and 2019.

### 2.2. Degree of Co-Occurrence among Colonizers at the Stand Level

**Figure 1:**
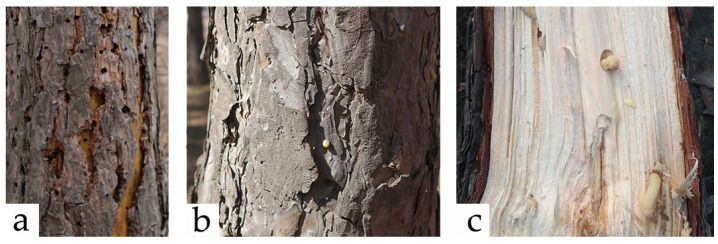
Emergence holes (**a**), resin beads (**b**), and larval tunnel (**c**) of *Sirex* woodwasps.

We observed exit holes and frass (Table 1) of co-colonizers within the lower 3 m of the trees. Binary data (yes/no) were collected for each dead tree regarding the presence of the four prevalent co-colonizers (*Sirex* woodwasps, *Asemum striatum* (Linnaeus, 1758), *Phaenops* sp. and *I. acuminatus*). Siricid circular exit holes can be distinguished from those of other woodborers (Table 1). Colonization by Siricid woodwasps can be determined for the current and preceding years, using two key diagnostic markers: resin beads for the current year and circular holes for the previous year (Figure 1). Dead/dying trees were marked using a handheld GPS (Global Positioning System, G310, Guangzhou Taike Electron Technology Co., Ltd., Guangzhou, China, accuracy < 3 m), and their diameter at breast height (DBH) and species (*P. sylvestris* var. *mongolica* or *P. tabuliformis*) were recorded.

### 2.3. Effect of Co-Occurrence on Woodwasps in Wood Segments

A total of 52 trees with resin beads on their bark were selected for felling in this experiment, comprising 40 from JBT and 12 from YS. Those included several infestation types: [*S. nitobei* only (X), *S. noctilio* only (S), co-occurrence (S + X)]. Every tree was sectioned into two parts (bottom and middle), each 200 cm in length, since *Sirex* species are rarely found in the upper sections [17]. Prior to felling, some bark beetles and woodborers emerged from the trees, leaving only a few hosts infested. Thus, the analysis the wood segments focused solely on the co-occurrence of the two woodwasps.

#### 2.3.1. Density of Woodwasps

The DBH of infested trees was measured to standardize estimates of insect density by wood volume. Quantifying density in the field is challenging due to the possibility of two woodwasps infesting the same tree, the fact that both types have identical exit holes and resin beads (Figure 1a,b), and the difficulty in distinguishing larval stage morphology (Figure 1c). Therefore, woodwasp species were confirmed based on morphological characteristics of the adults that emerged after rearing. We identified the species of emergence holes using two indices: the emergence periods of the two wasp species are relatively distinct, allowing for the differentiation of most adults based on the timing of emergence [17]. For the few adults with overlapping emergence periods, we marked the species and locations of emerging adults at different times each day to facilitate distinction. Emergence holes of *Sirex* species were then counted to compare densities and the spatial distribution of them.

#### 2.3.2. Body Size of Woodwasps

Significant variations in body size characterize the biology of dimorphic woodwasps [39]. We analyzed the correlation between pronotum width, ovipositor length, wing length, and body length (n = 100). Indices that were easy to measure, relatively stable, and highly correlated with body length were selected as criteria for evaluating body size.

### 2.4. Statistical Analysis

The percentages of emergence holes of *Sirex* woodwasps were evaluated using the nonparametric Kruskal–Wallis H test followed by Dunn’s test for post-hoc multiple comparisons among four sites. The presence (denoted as 1) or absence (denoted as 0) of colonized pests on each tree was recorded, with trees without pest damage being excluded. The phi coefficient, a measure of correlation between two dichotomous variables, was employed in SPSS 26.0.0 to examine the correlation between woodwasps and other pests (*A. striatum*, *I. acuminatus*, and *Phaenops* sp.). Statistical significance was determined using the chi-square test. Ripley’s *K*-function with border correction for spatial inhomogeneity [40] was employed to assess the distribution of co-colonizing populations within trees. Based on the two-dimensional point coordinate distribution of different populations, the spatial distribution patterns of individual colonizer populations were quantitatively analyzed. This method tests complete spatial randomness (CSR) by employing an extended search radius centered on each target tree. The maximum search radius was set to 15 m. Point pattern analysis was conducted using R Studio (version 2024.04.2-764. for Windows) and the ‘spatstat’ package (version 3.0-8). A 95% confidence envelope was calculated through Monte Carlo random simulation of 299 bootstrapping replications. Aggregation is indicated by space above the envelope, while regularity is indicated by space below it. Dead tree samples from field surveys, encompassing data from the current year (y) and the previous year (y − 1), were analyzed as response variables, and a spatial lag model was constructed. All data were processed using ArcGIS version 10.8 and analyzed with GeoDa version 1.22.0.4.

Due to the non-significant interaction between host sections and infestation types, separate analyses were performed for the bottom and middle sections of the wood segment. Considering the normality of the data and homogeneity of variances, unpaired t-tests, Welch’s t-test, or Mann–Whitney tests were conducted for assessing the body size and density of wood wasps (GraphPad Prism version 10.0.0 for Windows). To evaluate changes in the distribution patterns of two woodwasp species, their colonization percentages within the mid-section of trees were compared.

## 3. Results

### 3.1. Colonizer Damage at the Stand Scale

**Figure 2:**
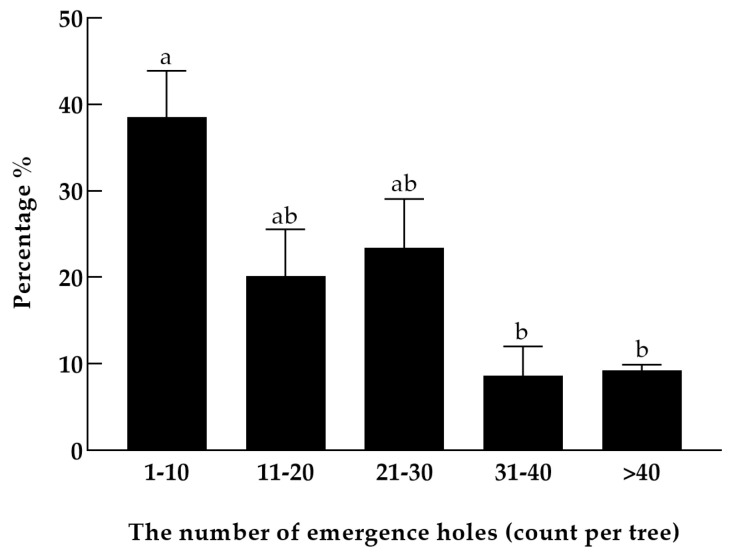
Density for emergence holes of *Sirex* woodwasps in damaged *Pinus sylvestris* var. *mongolica*. a, b: *p* < 0.05.

The emergence-hole diameters (*S. noctilio*: 3.89–6.91 mm; *S. nitobei*: 3.04–5.63 mm) and near emergence-hole orientation (Figure S1) (n = 40 *S. noctilio*: 15% upward, 85% downward; n = 40 *S. nitobei*: 70% up, 30% down) partially overlapped between species. Therefore, our statistics did not allow for accurate differentiation of the damage characteristics between the two woodwasp species. Consequently, spatial distribution analysis at the stand level was confined to the genus level. The majority of damage to *P. sylvestris* var. *mongolica* was attributed to several colonizing agents, including *Sirex* woodwasps, *A. striatum*, *I. acuminatus*, and *Phaenops* species. Overall, the complex damage caused by colonizers across various genera amounted to 1.45%. *Sirex* woodwasps alone accounted for damage in up to 12.32% of host trees. At the four study sites, 82.75% of the trees affected by woodwasps exhibited 1 to 30 emergence holes at standard visual height (Figure 2).

**Figure 3:**
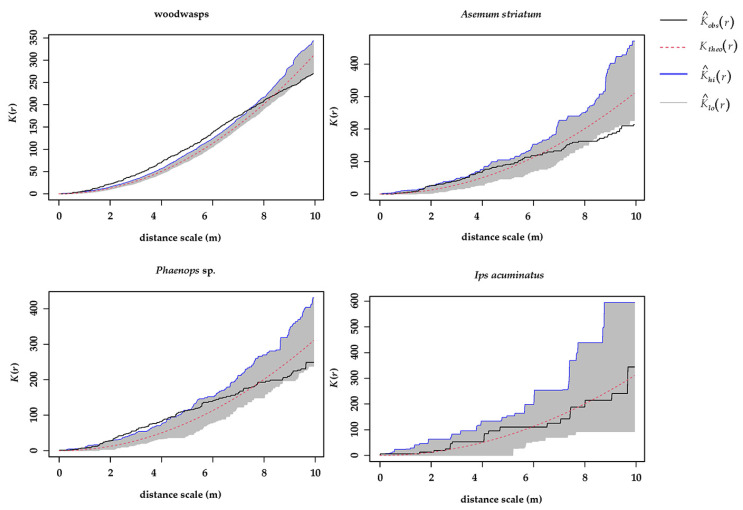
Spatial distribution of woodwasps and other woodborers based on Ripley’s *K* function. Red line: completely random spatial distribution. Gray area indicates a 95% confidence. Black line: degree of aggregation; Upper gray areas: clustered, Lower part: uniform distribution.

Ripley’s *K* function analysis revealed that colonizing agents exhibited spatial aggregation within a moderate spatial scale, specifically within a radius of 0–8 m. *Sirex* woodwasps demonstrated clustering within a distance range of 0–7.3 m, with their distribution becoming random beyond 7.3 m. The highest aggregation degree of *Sirex* occurred at a radius of 4.1 m. Conversely, the distribution of *A. striatum* and *I. acuminatus* appeared random within the 0–8 m radius. *Phaenops* sp. exhibited the highest concentration within a 3.1 m radius (Figure 3).

### 3.2. Within-Tree Associations at the Tree Scale

This study analyzed a total of 276 dead trees with substantial colonizer damage. *Sirex* woodwasps were negatively associated with most colonizing agents (*p* < 0.001), except for *I. acuminatus*, with which they maintained a neutral association (*p* > 0.05). *Phaenops* sp. exhibited a positive relationship with both *A. striatum* and *I. acuminatus* (*p* < 0.001), and it demonstrated neutrality between *A. striatum* and *I. acuminatus* (Table 2).

The autologistic regression model revealed a significant increase in the frequency of *Sirex* woodwasp attacks with attack density of *Sirex* over two years, density of *Phaenops* sp. attacks from the previous year (y − 1), and *A. striatum* attack density from the current year (Table 3). The presence of *Phaenops* sp. in the current year and *A. striatum* in the previous year were negatively associated with *Sirex* woodwasp attacks. The influence of *I. acuminatus* on woodwasp attacks was not statistically significant (*p* > 0.05).

### 3.3. Effect of Co-Occurrence on Woodwasps in the Wood Segment

#### 3.3.1. Density of Woodwasps

**Figure 4:**
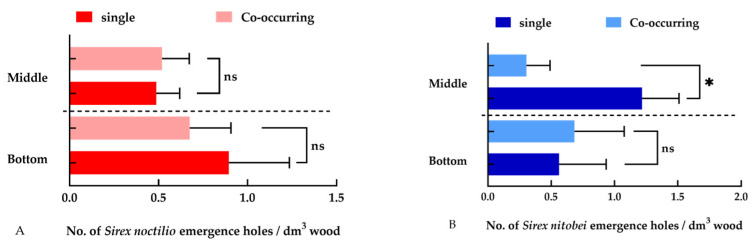
Mean density of emergence holes for (**A**) *Sirex noctilio* and (**B**) *S. nitobei* in the bottom and middle sections of trees co-inhabited by both species, as well as in trees inhabited by only one species. Error bars represent the SE, *: *p* ≤ 0.05; ns: *p* > 0.05.

We felled 52 trees (including the bottom (0.7–2.7 m) and middle tree sections (2.7–4.7 m)) to assess the density of woodwasps. In total, 1172 emergence holes of *S. noctilio* and 782 of *S. nitobei* were counted. We found that 65 ± 3.04% (*p* < 0.01) of *S. nitobei* emerged from S + X tree sections, while 59 ± 1.59% of *S. noctilio* emerged from S tree sections. The density of *S. noctilio* did not significantly differ in either the bottom (0.89 ± 0.34 vs. 0.68 ± 0.23 dm^−3^, F = 1.836, *p* = 0.7078) or middle (0.49 ± 0.13 vs. 0.52 ± 0.15 dm^−3^, F = 1.956, *p* = 0.3205) tree sections (Figure 4A). In the middle section, the density of *S. nitobei* in trees with both S and X was significantly lower than in X trees (F = 5.463, *p* < 0.05) (Figure 4B). In the bottom section, the density was not significantly affected (F = 1.614, *p* = 0.5816). There was a general increasing trend in the bottom section of S + X trees (0.68 ± 0.39 dm^−3^).

#### 3.3.2. Body Size of Woodwasps

**Figure 5:**
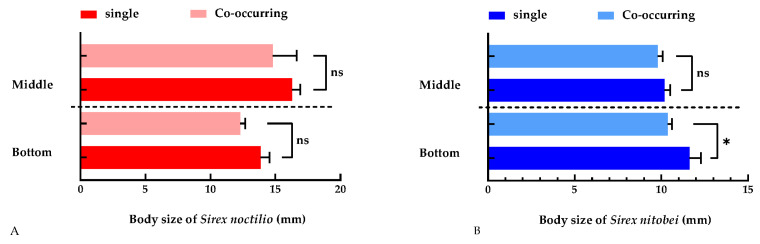
Mean body size of (**A**) *Sirex noctilio* and (**B**) *S. nitobei* in the bottom and middle sections of trees co-inhabited by both species and those inhabited by only one species. Error bars represent the SE, *: *p* ≤ 0.05; ns: *p* > 0.05.

Given the ease of measurement and the strong correlation with body length (ranging from 0.87 to 0.97), wing length was ultimately employed as an indicator of body size. There was no significant difference in the body size of *S. noctilio* in S + X trees compared to S trees, both in the bottom (14.79 ± 1.84 mm vs. 12.29 ± 0.40 mm, F = 2.314, *p* = 0.0554) and middle (16.26 ± 0.64 mm vs. 13.86 ± 0.69 mm, F = 1.559, *p* = 0.3847) tree sections (Figure 5A). In contrast, *S. nitobei* was larger in X trees (11.62 ± 0.66 mm vs. 10.36 ± 0.26 mm, F = 1.567, *p* < 0.05) compared to S + X trees in the bottom tree section. No significant difference was observed in the body size of *S. nitobei* between S + X and X trees (10.18 ± 0.32 mm vs. 9.78 + 0.29 mm, F = 2.314, *p* = 0.4190) in the middle section (Figure 5B).

### 3.4. Within-Tree Distribution of Woodwasps in Different Infestation Types

**Figure 6:**
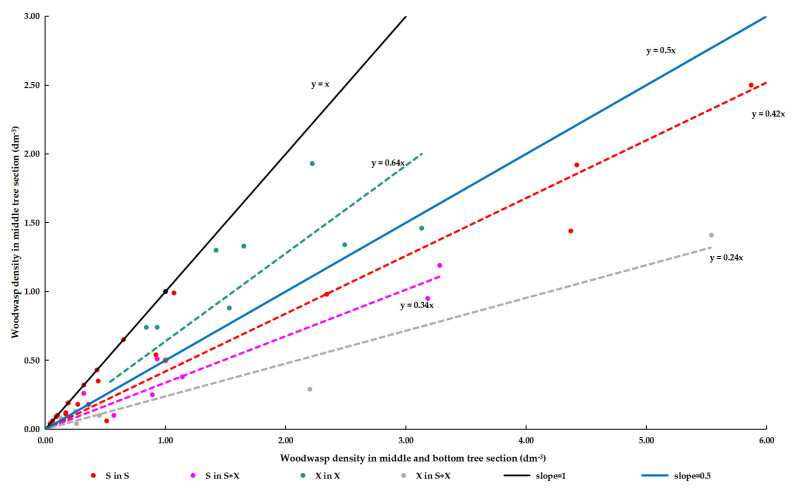
Distribution of *Sirex noctilio* and *S. nitobei* within *Pinus sylvestris* var. *mongolica* trees in Northeast China. The distribution patterns are illustrated as follows: *S. noctilio* in trees with both S and X (light rose red line), *S. noctilio* in trees with S only (red line), *S. nitobei* in trees with both S and X (grey line), *S. nitobei* in trees with X only (green line), slope = 0.5 (blue line), and slope = 1.0 (black line). S: *Sirex noctilio*; X: *Sirex nitobei*; S + X: *Sirex noctilio* and *S. nitobei*.

The spatial distribution of both *Sirex* species within trees varied among S, S + X, and X trees (Figure 6). *Sirex noctilio* exhibited a more concentrated distribution in the bottom section in both S (95% CI = 0.38–0.46) and S + X (95% CI = 0.28–0.40) trees. In X trees, *S. nitobei* was more concentrated in the middle section (95% CI = 0.48–0.80), whereas it was more abundant in the bottom section of S + X trees (95% CI = 0.19–0.28).

## 4. Discussion

Research on the co-occurrence patterns of invasive and native species can help elucidate and clarify their potential interactions. The field survey revealed co-occurrence of *S. nitobei*, *A. striatum*, *Phaenops* sp., and *I. acuminatus* with *S. noctilio*. Across spatial scales, neutral (*I. acuminatus*) and negative (*A. striatum* and *Phaenops* sp.) associations were observed between *Sirex* woodwasps and other co-occurring insects. The emergence period of *I. acuminatus* in the Inner Mongolia region spans from May to July, relatively preceding the eclosion of two woodwasp species. Studies indicate that ophiostomatoid fungi exhibit potent repellent properties against unmated female woodwasps [41]. As a result, later-emerging woodwasps typically avoid *Ophiostoma clavatum* (Mathiesen-Käärik) Hunt, a fungus associated with *I. acuminatus*, during oviposition site selection. This behavior mitigates the antagonistic impact of ophiostomatoid fungi on woodwasp symbionts, thus facilitating a conducive environment for the growth of these symbiotic fungi and the development of their offspring [42]. This likely explains the neutral association between the beetle and the woodwasp. *Asemum striatum* has an extended flight period, and the early-emerging adults can utilize the weakened wood, following woodwasp attacks, to supplement their nutrition in the current year. Once winter sets in, the growth environment for the late-emerging individuals deteriorates, hindering their development. Trees severely damaged by *A. striatum* typically weaken and die within a year, which is not conducive to woodwasp egg-laying and development. Consequently, a positive correlation was observed between woodwasps and *A. striatum* in the current year, whereas a negative correlation was detected in the previous year. In North America, mixed associations were also observed between *S. noctilio* and woodborers [33]. *Sirex noctilio* has a net positive effect on *Pissodes* sp. (arrived after *S. noctilio*) population densities at a landscape scale in North America [43] and a weakly positive association with *Tomicus piniperda* (Linnaeus) and Cerambycidae in Spain [44]. Co-habiting beetles could have a negative impact on the population dynamics of *S. noctilio* over time [26,29,45]. Mixed associations observed between them may be attributed to the differences in host physiological state preferences among different species.

Studies have shown that *S. nitobei* and *S. noctilio* exhibit overlap in their preferences and behavior towards declining or recently dead trees [13,17,19,24,38,43]. Consequently, these species notably aggregate to share resources. Interspecific competition between them is relatively weak [17], as the population density of woodwasps is low compared to outbreaks in the Southern Hemisphere which can reach 50–100 individuals per meter of log [46]. This study suggests a minimal effect of *S. nitobei* on *S. noctilio*. The ability of *S. noctilio* to kill healthier trees at higher densities [22,47,48] likely creates additional habitat for *S. nitobei* in trees that would otherwise be too healthy and unavailable. We hypothesize that *S. noctilio* may have a net positive effect on *S. nitobei*, as its presence appears to enhance *S. nitobei* numbers at the stand level, necessitating additional investigation. Research indicates a positive correlation between the body size of *Sirex nigricornis* Fabricius and DBH of their host trees [49], a relationship not observed in *S. noctilio* and *S. nitobei* in this study. Consequently, the impact of DBH on woodwasp body size was not considered in the analysis. Ryan et al. (2012) suggested a negative correlation between body size and density [45]. Smaller (but higher densities of) *S. nitobei* emerged from the bottom part of S + X trees compared to X trees. The body size of *S. noctilio*, which generally arrive first [17], seems unaffected by *S. nitobei*. The density of woodwasps within a forest stand may lead to differences in inter-landscape-scale interactions. Therefore, *S. noctilio* may have both positive (density) and negative (body size) impacts on *S. nitobei* in current study. Similarly, *Pissodes* sp. (arrived after *S. noctilio*) had no effects on the body size of *S. noctilio* [43]. Conversely, a larger body size was observed in *S. noctilio*, especially in males, within beetle-infested trees in Canada [45]. Ryan et al. (2012) suggested that the accelerated drying of sapwood, induced by ophiostomatoid fungi [50], promotes the growth of symbiotic fungi [51], enhancing larval nutrition in beetle-infested trees, which may explain the phenomenon [45]. The emergence period appears to be a significant factor influencing the body size of woodwasps.

This study identified the degree of co-occurrence of *S. noctilio* and *S. nitobei* in northeastern China. Ignoring the influence of other colonizers (trees with only resin beads), the changes in the spatial distribution of *S. nitobei* within trees potentially indicate interactions between the two species of woodwasps. This suggests that the presence of *S. noctilio* influences the distribution of *S. nitobei* within the tree, indicating resource partitioning in the current year [52,53,54]. In trees infested with both *S. noctilio* and *S. nitobei* (S + X), a greater proportion of *S. nitobei* was found in the lower sections compared to trees infested only with *S. nitobei* (X). Consequently, *S. nitobei* aggregation and dispersal patterns, as well as egg-laying preferences, seem to be influenced by *S. noctilio*, particularly in the lower sections of infested trees with higher insect densities and more emergence holes [44,55,56]. *Sirex noctilio* predominantly colonizes in the bottom section (S + X or S) of the tree, irrespective of the presence of *S. nitobei*. This is consistent with the oviposition habits of *S. noctilio* observed in multiple countries, including in the Southern Hemisphere, such as South Africa, New Zealand, Australia, Brazil, and in the Northern Hemisphere, such as Canada [44,55,56,57]. In the autumn of the current year, native woodwasps do not appear to avoid trees already colonized by *S. noctilio* within the stands, according to our findings. *Xeris* species do not form a symbiotic relationship with fungi; instead, they deposit eggs in trees that have been previously attacked by other siricid species and infected with their fungi [58]. In North America, *Xeris* species may feed on *A. areolatum* or *A. chailletii*. *Xeris spectrum* Linné, which lacks a symbiotic fungus, can utilize the fungal symbionts of *S. nitobei* [58,59]. During summer, *X. spectrum* adults emerge with woodwasps carrying fungi, laying their eggs on trees already colonized by fungi. In the subsequent spring, they deposit eggs on trees previously infected with fungi [59,60,61]. These findings suggest that horizontal transmission of interspecies symbiotic fungi between siricid woodwasps may be enhanced, regardless of whether the woodwasp carries the fungus [12]. This can occur when fungi are acquired from the environment, potentially complicating pest management strategies.

## 5. Conclusions

Random sampling was undertaken in *P. sylvestris* var. *mongolica* plantations to evaluate insect co-colonization patterns and extents, revealing potential interactions across different spatial scales. At the stand scale, *Sirex* woodwasps represented negative associations with *A. striatum* and *Phaenops* sp., and neutral associations with *I. acuminatus*. Clustering of woodwasps and *Phaenops* sp. were observed at a small scale. The degree of woodwasp damage is most affected by the degree of woodwasp damage in the current and previous year, with other colonization factors having minor impacts. The availability of host resources and the spatiotemporal niche differentiation among colonizers may explain their lower co-occurrence rates and decreased interspecific interactions.

Analyzing spatial patterns within trees provides insights into the extent and degree of resource partitioning between the two woodwasps. *Sirex noctilio* was more abundant in trees where it occurred alone. The distribution and body size of *S. noctilio* within the trees appeared unaffected by *S. nitobei*. In the presence of *S. noctilio*, *S. nitobei* tended to lay eggs in the damaged sections of the trees. This can be attributed to early *S. noctilio* attacks enhancing the micro-environment of *S. nitobei*, thereby providing nutrients for larval development. At the stand level, the overall impact of *S. noctilio* on *S. nitobei* population density is likely positive because *S. nitobei* prefer weaker trees, a preference potentially influenced by initial attacks from *S. noctilio* on healthier hosts.

## Figures and Tables

**Table 1:** Damage characteristics of *Sirex* woodwasps and other bark- and woodboring insects.

Characteristics	*Sirex* Woodwasps ^1^	*Asemum* *striatum*	*Phaenops* sp.	*Ips* *acuminatus*
Shape of emergence hole	Circle	Ellipsoid	Similar to the letter “D”/half-moon	Circle
Diameter of emergence hole	3.04–6.91 mm	4.00–13.00 mm	≈3 mm	1–2 mm
Treatment of frass	Frass accumulates in the gallery	Frass discharged from the gallery	Frass accumulates in the gallery	Frass fills the gallery

^1^: Characteristics of native *Sirex nitobei* are indistinguishable from *S. noctilio* based on our statistics.

**Table 2:** Phi (φ) coefficients of colonizers on 276 dead trees.

	*Asemum striatum*	*Phaenops* sp.	*Ips acuminatus*
*Sirex* woodwasps	−0.297 ***	−0.291 ***	−0.116
*Asemum striatum*		0.270 ***	0.076
*Phaenops* sp.			0.214 ***

*** *p* < 0.001.

**Table 3:** Spatial lag model testing effects of colonizers from the current year (y) and the previous year (y − 1).

Year	Variable	Coefficient	SE	z-Value	Probability
y − 1	*Sirex* woodwasps	0.993	0.006	172.67	***
	*Asemum striatum*	–0.442	0.058	−7.59	***
	*Phaenops* sp.	0.461	0.068	6.78	***
	*Ips acuminatus*	–0.041	0.036	−1.17	0.24
y	*Sirex* woodwasps	0.991	0.006	163.88	***
	*Asemum striatum*	0.443	0.058	7.63	***
	*Phaenops* sp.	–0.437	0.070	−6.25	***
	*Ips acuminatus*	0.018	0.016	1.12	0.26

*** *p* < 0.001.

## Data Availability

The data are available from the authors upon request.

## Supplementary Materials

The following supporting information can be downloaded at: https://www.mdpi.com/article/10.3390/insects15070478/s1, Figure S1. Woodwasp emergence hole orientation in longitudinal section of the damaged *Pinus sylvestris* var. *mongolica*. Emergence holes of the two *Sirex* species had both upward and downward orientation.
