# Supplementary figures and images for: Resource Sharing between the Invasive Sirex noctilio and Native Woodborers and Beetles in Pinus Plantations
Source: Insects. 2024 Jun 27;15(7):478. doi: 10.3390/insects15070478 (PMC11277501; doi:10.3390/insects15070478)

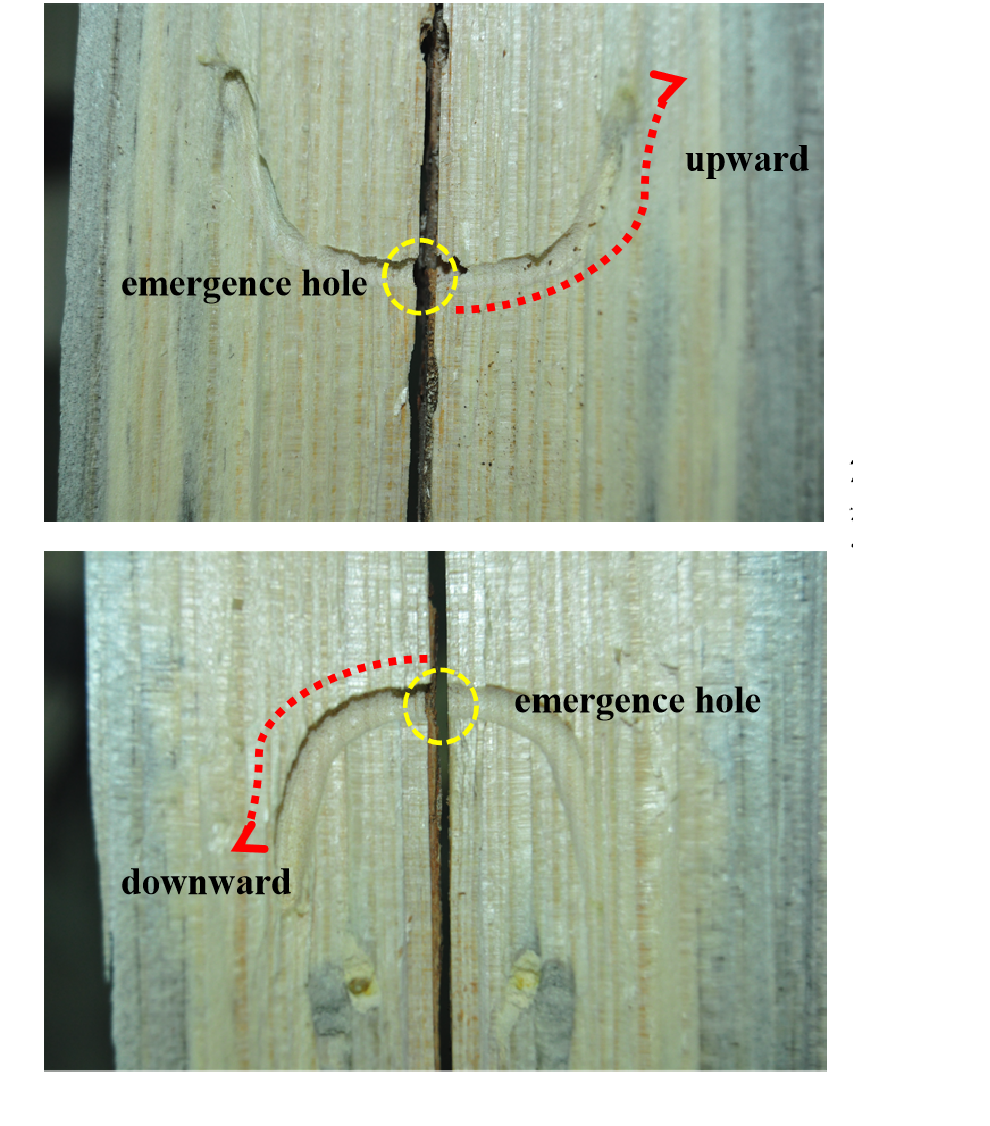

Supplement: Supplementary file 1 [file insects-15-00478-s001.zip › insects-3055129-supplementary.tif]
